# Automatic imitation is modulated by stimulus clarity but not by animacy

**DOI:** 10.3758/s13414-024-02935-1

**Published:** 2024-07-31

**Authors:** Hannah Wilt, Yuchunzi Wu, Antony Trotter, Patti Adank

**Affiliations:** 1 Department of Speech, Hearing and Phonetic Sciences, University College London, London, WC1N 1PF UK; 2 Department of Neural and Cognitive Sciences, New York University Shanghai, Shanghai, China; 3 NYU-ECNU Institute of Brain and Cognitive Sciences at New York University Shanghai, Shanghai, China; 4 Institute of Psychiatry, Psychology & Neuroscience, King’s College London, London, UK

**Keywords:** Automatic imitation, Animacy, Visual clarity, Manual actions, Stimulus response compatibility

## Abstract

Observing actions evokes an automatic imitative response that activates mechanisms required to execute these actions. Automatic imitation is measured using the Stimulus Response Compatibility (SRC) task, which presents participants with compatible and incompatible prompt-distractor pairs. Automatic imitation, or the *compatibility effect*, is the difference in response times (RTs) between incompatible and compatible trials. Past results suggest that an action’s animacy affects automatic imitation: human-produced actions evoke larger effects than computer-generated actions. However, it appears that animacy effects occur mostly when non-human stimuli are less complex or less clear. Theoretical accounts make conflicting predictions regarding both stimulus manipulations. We conducted two SRC experiments that presented participants with an animacy manipulation (human and computer-generated stimuli, Experiment 1) and a clarity manipulation (stimuli with varying visual clarity using Gaussian blurring, Experiments 1 and 2) to tease apart effect of these manipulations. Participants in Experiment 1 responded slower for incompatible than for compatible trials, showing a compatibility effect. Experiment 1 found a null effect of animacy, but stimuli with lower visual clarity evoked smaller compatibility effects. Experiment 2 modulated clarity in five steps and reports decreasing compatibility effects for stimuli with lower clarity. Clarity, but not animacy, therefore affected automatic imitation, and theoretical implications and future directions are considered.

**Supplementary information:**

The online version contains supplementary material available at 10.3758/s13414-024-02935-1.

## Introduction

Observing actions activates neural and cognitive mechanisms required to execute these actions (Buccino et al., 2004; Fadiga et al., 1998). Such covert imitation, or *automatic imitation,* is measured using the Stimulus Response Compatibility (SRC) task. In a manual SRC task, participants perform a finger or hand movement in response to a prompt (e.g., lift index finger upon seeing ‘£’) (Brass et al., 2000). The prompt is usually superimposed on a distractor image or video of a hand lifting its index or middle finger. When the prompt is presented in the presence of a compatible distractor (‘£’ with a video of a lifting index finger), participants are faster to perform the correct response than when the prompt is presented with an incompatible distractor (‘£’ with a lifting middle finger video). For compatible distractors, action observation is thought to facilitate motor patterns required for performing the prompted action, thus reducing response times (RTs) and accuracy. In contrast, incompatible distractors evoke competition between these facilitated motor patterns and the patterns required to produce the prompted response, leading to longer RTs and reduced accuracy. More automatic imitation, or a larger *compatibility effect*, measured as the RT difference between incompatible and compatible pairs, is interpreted as that motor mechanisms were more facilitated for the compatible distractor and/or more inhibited for the incompatible distractor. SRC paradigms are thought to provide a direct measure of the relative activation of motor patterns, and therefore of covert imitation (Heyes, 2011).

A comprehensive meta-analysis (Cracco et al., 2018) proposed that automatic imitation results from learning sensorimotor associations (Heyes, 2011; Heyes & Catmur, 2022). Cracco et al. suggested that automatic imitation is driven by domain-general mechanisms and arises mainly from movement and effector compatibility. The authors concluded that only a single social factor, namely similarity between actor (i.e., the distractor stimulus) and imitator (the participant), modulates automatic imitation. Similarity between actor and imitator has thus far been implemented by either manipulating the biological status, or *animacy,* of the actor executing the distractor stimulus, or the physiological plausibility of the distractor stimulus. For instance, several studies created distractor stimuli consisting of moving robotic fingers, hands, or arms, or computer-generated voices (Bird et al., 2007; Brass et al., 2001; Jansson et al., 2007; Kilner et al., 2003; Kupferberg et al., 2012; Liepelt & Brass, 2010; Liepelt et al., 2010; Oztop et al., 2005; Press et al., 2005, 2006; Wilt et al., 2022).

Current automatic imitation accounts consider effects of animacy modulations from two perspectives. *Self-other overlap* accounts (Brass & Heyes, 2005; Cook et al., 2014; Greenwald, 1970; Heyes, 2005, 2011; Prinz, 1997) explain decreases in automatic imitation in terms of the strength of sensorimotor associations between observed distractor stimuli and the response. The observation of distractor stimuli produced by similar actors (e.g., humans) is predicted to elicit stronger compatibility effects than those produced by dissimilar actors (i.e., non-human agents). Others, referred to here as *motivational* accounts (Chartrand & Dalton, 2009; Wang & Hamilton, 2012), explain animacy effects on the basis that people consciously or sub-consciously control imitation in a ‘Machiavellian’ way to affiliate with others. Thus, people are predicted to imitate more if they affiliate more with an agent, and as people are expected to affiliate more with human than with non-human agents, more automatic imitation is expected for human than for non-human stimuli. People have been reported to imitate more when they have a goal to affiliate (or get along well) with others. For example, in Lakin and Chartrand (2003), participants who received an affiliation goal (i.e., they were instructed to get along well with a stranger) in an experimental setting copied behaviours more than those participants who received no affiliation goal. Imitation was measured as the number of times a participant touched their face after the experimenter touched her face while participants completed a visual distractor task. Both accounts thus predict that observers will show more automatic imitation for human than for non-human stimuli, although they presume this effect originates from different cognitive mechanisms. However, it is unclear how past results from studies manipulating animacy support this prediction, as their results are mixed with respect to effects of animacy manipulations on automatic imitation.

### Animacy manipulations

Brass et al. (2001), Kilner et al. (2003), Press et al. (2005) and Bird et al. (2007) support the prediction from the self-other overlap and motivational accounts, as they report enhanced automatic imitation for human compared to non-human distractors. In Brass et al.’s (2001) Experiment 1, participants completed an SRC task in which they executed a finger-movement task in the presence of human distractor stimuli depicting compatible and incompatible finger movements. Participants performed a prespecified response per stimulus block, namely lifting their index finger (lifting) or moving the finger down (tapping). They were instructed to move their finger as soon as they detected movement in the video. Response times (RTs) were measured using electromyography (EMG) from participants’ right First Dorsal Interosseus (FDI) muscle. An overall compatibility effect of 33 ms (i.e., RTs for the compatible trials were 33 ms faster than for incompatible trials) was reported for Experiment 1, replicating Brass et al. (2000). In Experiment 2, participants were shown distractors consisting of human hands as in Experiment 1, as well as moving squares (non-human). Here, results showed a compatibility effect for the human distractors similar in magnitude to that reported for Experiment 1, but no compatibility effect for the non-human distractors.

Participants in Kilner et al. (2003) made arm movements while observing human or non-human distractors. The human distractors consisted of arm movements produced by a male human, while the non-human arm distractors were produced by a robot. Kinematic data were collected from three infrared markers placed on the participant’s right arm. In each condition, the participant was instructed to perform pre-specified sinusoidal vertical or horizontal movements with their right arm from the shoulder, while they observed blocks of incompatible or compatible human or non-human distractor videos. They were not given instructions regarding the distractors’ animacy. Enhanced compatibility effects were found for the human compared to the non-human distractors.

Press et al. (2005) presented participants with stimuli depicting an opening or closing hand. Animacy was implemented in a factorial design: the distractors were produced by a human or a robot (non-human) and were either naturalistic or schematic. RTs were measured using EMG from participants’ right FDI muscle. Participants made a prespecified response per stimulus block by either opening or closing their hand as soon as they detected movement in the distractor stimulus. Enhanced compatibility effects were found for human (33 ms) compared to non-human (6 ms) distractors and no difference between schematic and naturalistic manipulations was found.

Bird et al. (2007) presented observers with two types of distractors, namely a black-and-white image depicting an opening and closing human hand, plus opening and closing pincher-style robotic images. Participants performed a prespecified response in each response block (open or close hand as soon as movement could be detected in the distractor video). Responses were measured using EMG from the right FDI muscle. Bird et al. tested participants with and without autism spectrum disorder (ASD). The results showed an enhanced compatibility effects for human (48 ms) compared to non-human distractors (22 ms). This effect was likely driven by the ASD group, as no (statistically significant) difference in automatic imitation was reported between the human (39 ms) and non-human (24ms) distractors when only the non-ASD group was considered.

In contrast, others report null effects for animacy manipulations (Jansson et al., 2007; Kupferberg et al., 2012; Liepelt et al., 2010; Oztop et al., 2005; Wilt et al., 2022). Jansson et al. (2007) tested if the enhanced compatibility effects for human distractors reported by Brass et al. (2001), Kilner et al. (2003) and Press et al. (2005) could be replicated using simple symbolic (non-human) distractors. Jansson et al. recorded kinematic data from infrared markers placed on participants' left index fingernail. Experiment 1 aimed to replicate results from Brass et al. (2001) and used two simple response tasks. In one block, participants tapped their index finger when they detected a change in the stimulus, and in the other block they lifted their index finger. The distractors consisted of a stylised two-frame movie depicting a moving finger or pen. The second frame depicted the stimulus tilted upward or downward, at varying stimulus-onset asynchronies (SOAs), and participants responded as quickly to this change as they could. Participants completed two blocks, one with the finger distractors and one with the pen distractors. The results showed similar compatibility effects for the human (finger) and non-human (pen) distractors. Experiment 2 aimed to replicate the results from Press et al. (2005) and had a similar setup to that in Experiment 1, except here participants were required to close their hand when the stimulus changed, and in the other block they opened their hand. The human distractors consisted of a three-frame stimulus depicting an opening and closing hand viewed from the side serving. The non-human distractors consisted of pairs of dots moving apart or closer to mimic the motion of an opening and closing hand. The results showed similar compatibility effects for human and non-human distractors. Experiment 3 aimed to replicate the results from Kilner et al. (2003). It consisted of two conditions, picturing a moving ‘dot’ instead of an arm. Both conditions represented non-human distractors, but the movements were made by a pure sinusoidal wave (non-human) or by capturing the human kinematics generated by a human attempting to produce sinusoidal motion (human condition). Participants tracked the dot with their index finger. Before each trial, participants were told what movement they should make (horizontal or vertical). In incongruent trials, their task was to change direction in the orthogonal direction. Again, the results showed similar compatibility effects for human and non-human distractors. Therefore, Jansson et al. did not replicate the enhanced compatibility effects for human distractors reported in Brass et al. (2001), Kilner et al. (2003) and Press et al. (2005) using a tightly controlled stimulus design, and instead showed similar compatibility effects for human and non-human distractors. Jansson et al. suggested that the animacy effects in Brass et al. (2001), Kilner et al. (2003) and Press et al. (2005) might have been due to confounding differences between the distractor stimuli including complexity, confounded spatial and conceptual compatibility, and varying predictability.

Participants in Kupferberg et al. (2012) were instructed to perform horizontal or vertical arm movements while observing distractors of a human agent or of a humanoid robot, who executed congruent or incongruent arm movements showing pointing actions. The vertical and horizontal arm robot movements were produced with a quasi-human minimum-jerk velocity profile, which was defined as an artificial human-like movement speed pattern. This pattern was described in the mathematical model (Flash & Hogan, 1985) capturing the common kinematics or stereotyped patterns of muscle activations in voluntary human arm movements. This model implemented maximal smoothness of major qualitative and quantitative aspects of single-joint arm motions by assuming the minimised mean-square of the jerk. Jerk was mathematically described as the rate of change of the acceleration of the movement. The human movements were produced by pointing the arm horizontal or vertical with the index finger pointing. Kinematic responses were collected using an infrared tracker attached to the participant's right index finger. The results showed similar compatibility effects for distractors procured by the human and the robot. Further null results were also reported in Oztop et al. (2005) and Liepelt et al. (2010), and see Wilt et al. (2022) for null results for vocal distractor stimuli.

In summary, the literature review shows a mixed result of animacy manipulations on automatic imitation. It is not obvious what caused the discrepancy in the reported results, but it appears that studies that found an enhanced animacy effect used highly stylised non-human distractors with low visual complexity. For instance, Brass et al. used moving squares for the non-human condition versus images of a hand for the human condition. Press et al. (2005) used blue opaque outlines depicting both the human and non-human ‘hands’. In contrast, when non-human stimuli were matched closely to the human stimuli, no animacy effects were found (cf. Jansson et al., 2007; Kupferberg et al., 2012; Liepelt et al., 2010; Oztop et al., 2005; Wilt et al., 2022). The observed discrepancy in animacy effects between the studies discussed may be due to the use of stylised versus closely matched stimuli, or due to a lack of control of stimulus-driven factors.

Moreover, the mixed pattern in the results discussed raises the possibility that human stimuli did not evoke enhanced automatic imitation compared to non-human stimuli due to increased familiarity or affiliation, as predicted by the self-other and motivational accounts, but that non-human stimuli did not evoke (the same amount of) sensorimotor priming. It can be argued that visual and auditory stimuli in SRC tasks need to represent the sensorimotor information that was also available when the sensorimotor link between perception and action was originally learned (Heyes, 2011) to evoke the sensorimotor priming measured as automatic imitation. If the visual/auditory information in the distractors in SRC task deviates too much, for example, because the visual shape or motion no longer resembles the stored template, or is too visually distorted, sensorimotor priming, and thus automatic imitation, may be reduced. One example of stylistic representation of human actions leading to reduced visuomotor priming can be found in Kerzel and Bekkering (2000). Kerzel and Bekkering conducted a series of vocal SRC tasks that used video distractor stimuli of a human speaker mouthing the syllables ‘ba’ and ‘da’ and reported automatic imitation effects for all human-produced stimuli. However, in their Experiment 4, they replaced these syllables with stylised animations, i.e., two horizontal lines. For ‘ba’ distractors, the lines moved together and then apart, while for the /da/ distractors the lines only moved apart, thus simulating the movement pattern formed by the lips during articulation of each syllable. Participants completed an SRC task in which they responded to a visual prompt by saying ‘ba’ or ‘da’ in the presence of these two types of moving lines. No compatibility effects were found, presumably because the distractor stimuli were too visually impoverished to evoke visuomotor priming. Thus, there appears to be a link between complexity or visual clarity of the non-human stimuli and the presence or absence of animacy effects.

### The current study

We examined the possibility that published animacy effects (i.e., enhanced automatic imitation for human stimuli compared to non-human stimuli) were due to a lower sensorimotor priming capacity of non-human distractors. We tested if animacy modulates automatic imitation per the self-other overlap and motivational accounts, when considering the visual clarity of the distractor, by evaluating how two stimulus-driven factors, animacy and visual clarity, evoke sensorimotor priming. The study’s overarching aim was therefore to disentangle contributions of stimulus animacy and stimulus clarity to automatic imitation.

We manipulated distractor animacy by presenting participants with manual actions produced by a human hand and by a computer. We ensured that participants were aware that the computer-generated stimuli were not produced by a human actor and the human stimuli were produced by a human, so that participants shared the same belief. The second factor, Clarity, was implemented via a stimulus degradation manipulation, namely the reduction of visual clarity using Gaussian blur. Using Gaussian blur, it is feasible to degrade the stimulus materials without changing their shape or overall complexity. Gaussian blurring can be implemented using graphics or video-editing software, and applying this manipulation will reduce detail in an image. For instance, applying Gaussian blur to an image has been shown to negatively affect object perception speed and naming accuracy (Laws & Hunter, 2006; Takahshi & Watanabe, 2015). In Experiment 2, we evaluated the effect of reducing stimulus clarity in five steps using Gaussian blur for human stimuli only.

We conducted two online experiments in which we manipulated Animacy and Clarity (Experiment 1) in a factorial design, or parametrically modulated Clarity only (Experiment 2). We also included a two-alternative forced-choice (2AFC) classification task following the main SRC task in both experiments to evaluate any differences in how participants perceived the human and computer-generated stimuli (Experiment 1) and blurred and clear stimuli (Experiments 1 and 2). If predictions of the self-other and motivational accounts are correct, sensorimotor priming (and thus automatic imitation) was expected to be enhanced for human stimuli compared to non-human stimuli. However, if automatic imitation is largely driven by how much a distractor evokes sensorimotor priming, automatic imitation was expected to be similar for human and non-human stimuli, and to be enhanced only when visual clarity is better.

## Experiment 1

### Methods

#### Participants

Participants were recruited on Prolific (/prolific.co) and the experiment was hosted on Gorilla (/gorilla.sc). Per our preregistration, we used Bayes’ stopping rule to determine our sample size. We initially set our minimum sample size to 32; the number of participants required to fully counterbalance the design and the same number as tested in Wilt et al. (2022). BF_10_ > 3 was considered evidence in favour of the alternative hypothesis, and we considered BF_10_ < 0.2 as evidence in favour of the null hypothesis (Raftery, 1995). After collection of the minimum sample, we intended to calculate the BF10 based on model fit (Jarosz & Wiley, 2014) for a model containing the two-way interaction between compatibility and animacy and/or clarity versus one including only the main effects, also per our pre-registration. However, the resulting linear models failed to converge, rendering them uninterpretable. To account for this lack of convergence, we decided to double the minimum sample. In addition, due to an error when replacing participants, we accidentally over-recruited, and obtained a final sample of 65 participants (32 female; *M*_age_ = 25.77 years, *SD*_age_ = 3.54 years, range_age_ = 19–31 years). To obtain this final sample, 77 participants were recruited, of whom 12 were excluded: three did not provide responses in the SRC task, two failed the catch trials, four had error rates (ERs) of over 50% in one or more condition in the SRC task, one had an overall ER over 3 SDs from the group mean, and one participant scored 0% in the 2AFC identification task.

All participants were between 18 and 30 years of age and declared being native monolingual speakers of British English and resident in the UK at the time of the experiment. All stated having normal hearing and normal or corrected-to-normal vision, to be right-handed, and to not have any neurological or psychiatric disorders. Participants had completed a minimum of five studies on Prolific with an approval rate of ≥ 95%, and ran the study on a computer through Chrome. Upon completion of the task, participants were paid £2. The experiment was approved by the Research Ethics Committee of University College London (UCL) and conducted under Project ID #15,365.001.

#### Materials

**Fig. 1:**
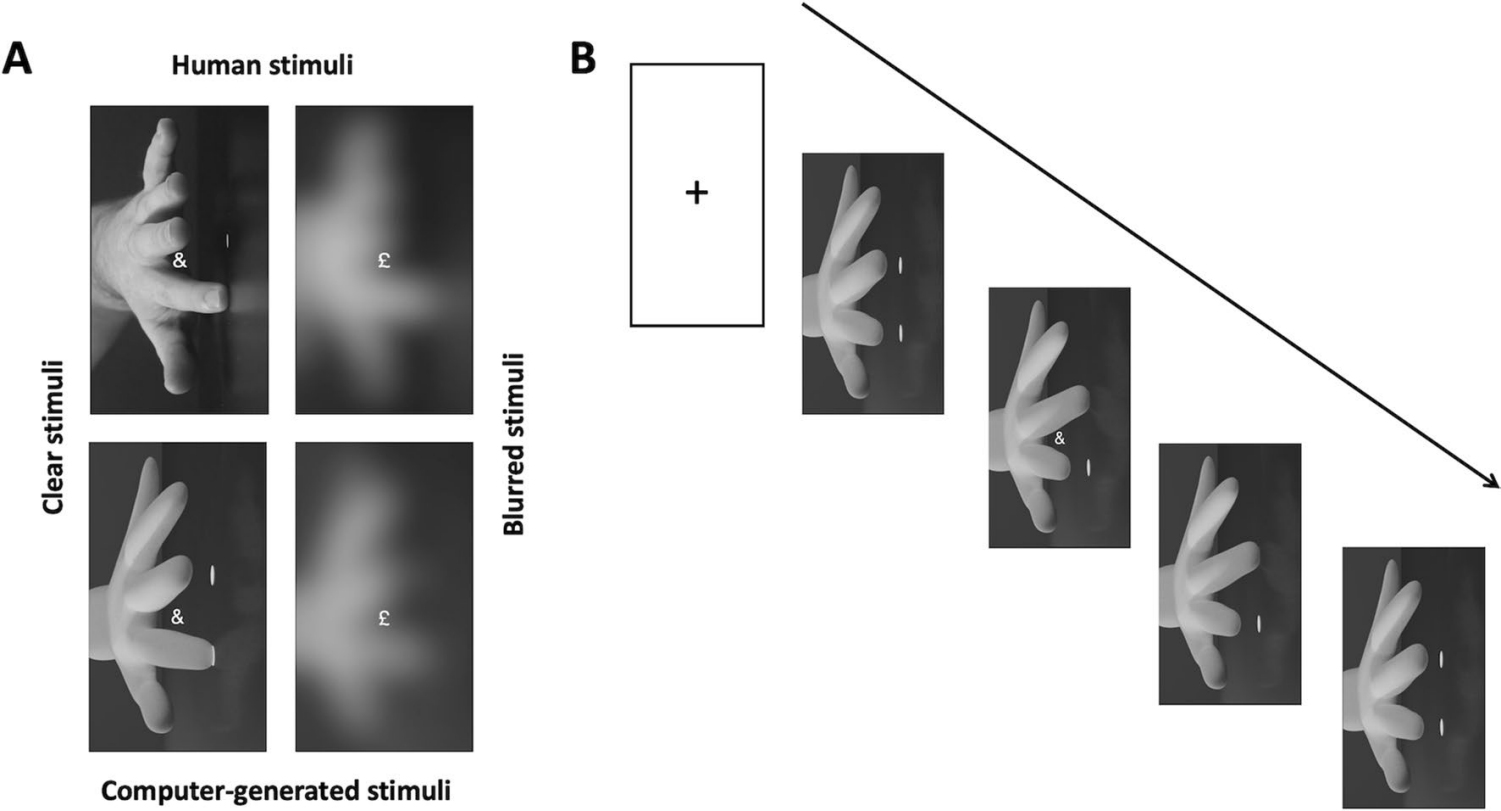
(**A**) Examples of the stimulus conditions (Animacy: human or computer-generated, and Clarity: clear or blurred). (**B**) Example of all events of one full trial in the experiment

The SRC video stimuli (50 fps,.mp4) were created on Final Cut Pro (Apple) using still images. The images depicted a clear or blurred human or a computer-generated hand (Fig. 1). The human hand stimuli were captured using a Canon Legria HF G30 video camera. The computer-generated hand stimuli were generated using Blender, and size and position were matched to the manual stimuli (www.blender.org). Next, the human and computer-generated stimuli were manipulated with the aim of decreasing their visual clarity. We used the Gaussian blur feature Final Cut pro, set to 50%, with the ‘blur boost’ level at 5, after a series of SRC pilot experiments (not presented here) to establish which settings effected a decrease in stimulus classification. For all four conditions (human/computer-generated and clear/blurred), images were generated depicting the hand in a neutral position, with all fingers lowered. Images were rotated 90° to minimise spatial compatibility effects (Catmur & Heyes, 2010). In each 3,000-ms video, the hand was first shown in a neutral position for 500 ms, followed by an image of either the index or the middle finger depressed for 1,000 ms, and finally the hand back in a neutral position for 1,500 ms. A response prompt (‘&’ or ‘£’) appeared in white Helvetica size 63 pt between the index and middle finger for 200 ms at 100 ms (SOA1) or 300 ms (SOA2) from the onset of the image with a finger lowered, i.e., 600 ms or 800 ms post video onset. The imperative prompt was integrated into the video directly rather than aligned on Gorilla (/gorilla.sc) to avoid stimuli-onset lags observed in online experiments (Bridges et al., 2020). See Fig. 1 for examples of all four stimulus conditions. For the 2AFC task, the same stimuli were used as in the SRC task except that symbol prompts were removed. For the catch trials, three images (.png) were created in Microsoft PowerPoint and saved as.tiff files in which an array of three, five or nine black dots appeared over a white background.

#### Procedure

Participants read the information sheet and provided consent. They then viewed detailed self-paced instructions for the stimulus–response compatibility (SRC) task, including prompt-response pairings. Prompt-response pairings were counterbalanced across participants. They were told they would observe distractor images produced by a human or generated by a computer programme. Participants were instructed to keep their right index finger over the left arrow key and right middle finger over the right arrow key, and to respond to the symbol prompt as quickly and accurately as possible. They viewed four sample trials, each followed by a description of the expected action response, as well as completing four practice trials. In the main task, participants completed four blocks of 32 trials (2 effectors × 2 prompts × 2 Animacy conditions × 2 Clarity conditions × 2 SOAs). Each block consisted of trials of all four conditions. Participants had the option of taking a short (1-min) break after each block. Each trial started with a 500-ms fixation cross over a white background, followed by 100 ms during which the fixation cross disappeared before the 3,000-ms SRC video was presented. Participants were reminded of the prompt-response pairings and instructed to answer as quickly and accurately as possible before each new block. A catch trial was included randomly within each block. Catch trials started with a 500-ms fixation cross, after which an array of dots (image) appeared for 750 ms. Participants indicated via button press whether they saw three, five or nine dots on the screen. They were to place their right hand appropriately above the arrow keys after responding to the catch trials and were reminded 500 ms after responding to do so before the onset of the next SRC trial. Participants were required to score ≥ 75% on the catch trials to be included in the final data analysis.

After the SRC task, participants completed a 2AFC task in which they were to identify the moving finger (index or middle) in each of the SRC stimuli. Each trial started with a 250-ms fixation cross that disappeared for 100 ms before playing the 3,000-ms video, which was identical to the videos used in the SRC task except that the symbol prompts were removed. Participants pressed the left arrow key to indicate that the stimulus depicted index finger tapping, and the right arrow key to indicate that the stimulus depicted middle finger tapping. They completed one practice trial and 32 task trials (4 × 2 effectors × 2 Animacy conditions × 2 Clarity conditions). This task was included to ensure the intelligibility of the effector in each video stimulus.

#### Data processing and analysis

Responses were recorded through the Gorilla.sc keyboard recording function. Response accuracy and response time (RT) from video onset were recorded online. Offline, RTs were corrected for time elapsed between video onset and prompts onset (600 ms for SOA1 and 800 ms for SOA2) to obtain RTs from prompt onsets. Erroneous responses, anticipatory answers (RT < 200 ms) and late answers (RT > 1,000 ms) were coded as errors. For the RT analyses, we removed errors as well as observations for which RTs were outside of three median absolute deviations (MADs) from a participant’s median RT in each condition (i.e., a combination of Animacy, Clarity, Compatibility, and SOA).

The data were analysed with generalised linear mixed effects models (GLMMs) using the *lme4* package in R (Bates et al., 2014). Separate models were run for the two dependent variables of interest, raw RTs (milliseconds) and errors (0 vs. 1). Fixed effects were Compatibility (compatible vs. incompatible, coded as -0.5 and 0.5, respectively), Animacy (human vs. computer-generated, coded as -0.5 and 0.5, respectively), Clarity (clear vs. blurred, coded as -0.5 and 0.5, respectively), SOA (SOA1 vs. SOA2, coded -0.5 and 0.5, respectively) and their interactions. For the RT analysis, we used the gamma distribution and identity link function following Lo and Andrews (2015). This type of link function is also considered preferable to transformation for RT data (Balota et al., 2013; Lo & Andrews, 2015; Schramm & Rouder, 2019). This analysis also allowed us to avoid potential issues reported with log-transforming and subsequently back transforming RT data (Feng et al., 2013; Lo & Andrews, 2015; Manandhar & Nandram, 2021; Molina & Martín, 2018). The accuracy analyses used a binomial distribution and logit link function. The maximal converging random effect structure for either set of models was employed, following Barr et al. (2013). For the RT analyses, this process included by-participant intercepts. For the error analyses, random effects consisted of random by-participant intercepts and by-participant slopes for Clarity. Backward selection was used to determine the best-fitting model. Starting with higher order interactions, predictors were removed one by one and chi-squared tests performed. A factor was removed from the model if it did not significantly improve model fit (*p* > 0.05) and if it was not included in any higher-order interactions. At each step, the factor for which there was least evidence of inclusion (i.e., highest p-value in the chi-squared test) was removed first and inclusion of the remaining factors re-assessed.

### Results

#### Reaction times (RTs)

The full dataset for 65 participants consisted of 8,264 observations. For the RT analyses, erroneous trials were removed (648 trials, 7.84%), consisting of 330 wrong answers, seven anticipatory responses (RT < 200 ms) and 311 late responses (RT > 1,000 ms). We further removed 613 observations with RTs outside of three MADs from each participant’s mean in each experimental condition (i.e., each combination of Compatibility, Animacy and Clarity). The remaining 7,003 trials were included in the RT analysis. The backward selection procedure from the saturated model and the descriptive statistics are given Appendix A, Tables A1 and A2. The final model (Table 1) included all main effects (Compatibility, Animacy, Clarity and SOA) as well as the two-way interactions between Compatibility and Clarity, and between Animacy and Clarity.

**Table 1:** Final model of reaction times (RTs) in milliseconds (ms) using a gamma distribution and identity link function

Fixed effect	Estimate	*SE*	t-value	p-value
**(Intercept)**	**572.608**	**6.301**	**90.877**	** < 2 × 10**^–16^***
**Compatibility**	**5.553**	**1.082**	**5.132**	**2.87 × 10**^–7^***
**Clarity**	**7.263**	**1.087**	**6.682**	**2.35 × 10**^–11^***
Animacy	1.398	1.077	1.298	0.194
**SOA**	**-10.069**	**1.091**	**-9.233**	** < 2 × 10**^–16^***
**Compatibility x Clarity**	**-2.745**	**1.085**	**-2.529**	0.011*
**Clarity x Animacy**	**3.029**	**1.094**	**2.760**	0.006**

*SOA* stimulus-onset asynchrony

^*^ *p* < .05, **p < .01, ***p < .001

**Fig. 2:**
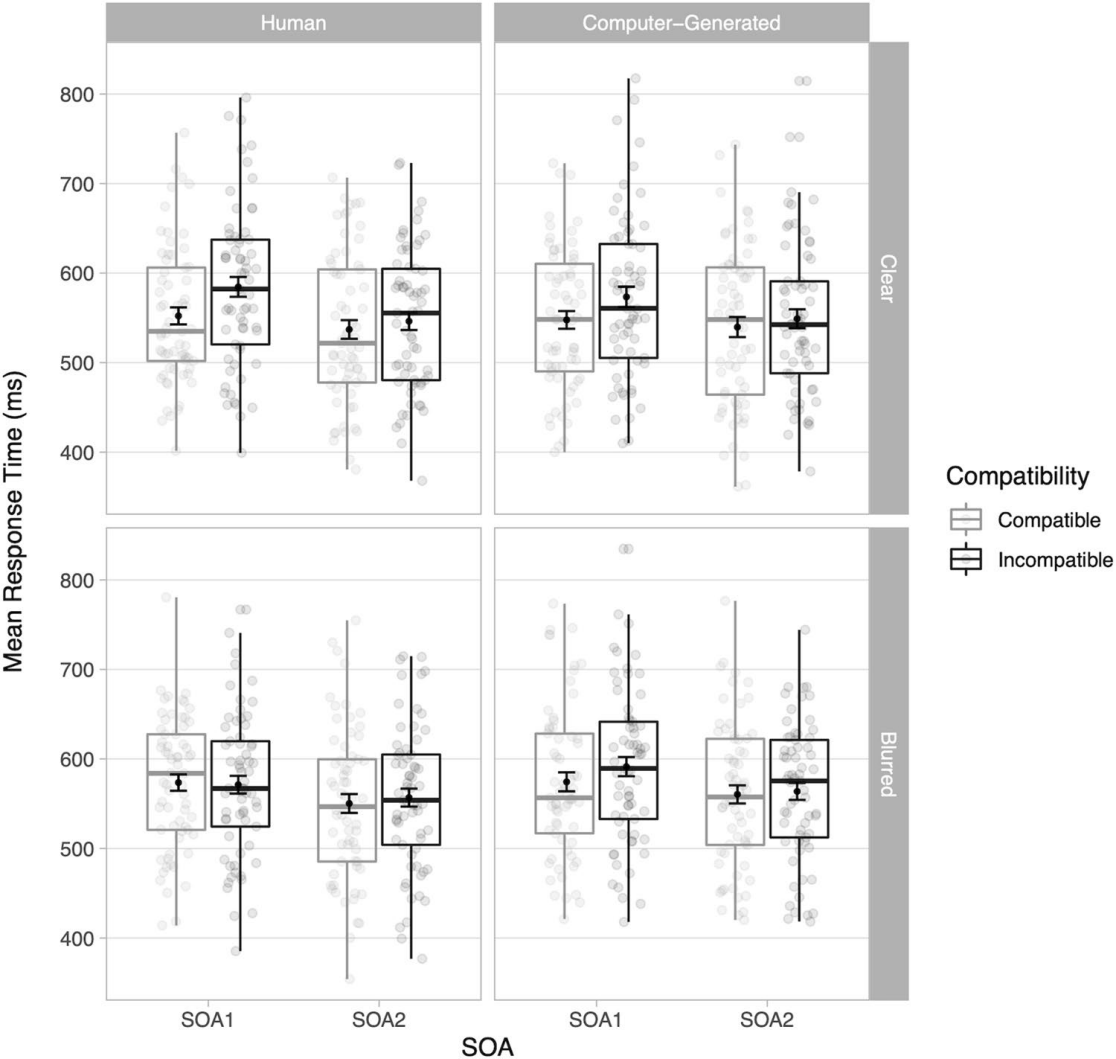
Mean response times (RTs) in milliseconds (ms) for correct trials in the stimulus–response compatibility (SRC) tasks for each experimental condition and stimulus-onset asynchrony (SOA). Points in the background show the raw mean RTs for each participant (points are offset on the x-axis for clarity). The boxplots indicate the first, second (median) and third quartiles, and whiskers indicate 1.5 times the interquartile range of the distribution. Black points in the foreground show the mean and error bars indicate standard errors

The main effect of Compatibility was significant, as RTs were slower for incompatible (*M* = 567 ms, *SD* = 85 ms) than for compatible trials (*M* = 554 ms, *SD* = 83 ms) (Fig. 2, Table 1), *d* = 0.15, indicating an overall compatibility effect of 13 ms. The main effect of Animacy was not significant, therefore showing no evidence that participants responded differently to human and computer-generated stimuli. The main effect of Clarity was significant, with slower RTs in response to blurred stimuli (*M* = 568 ms, *SD* = 82 ms) than to clear stimuli (*M* = 554 ms, *SD* = 86 ms), *d* = 1.67. The main SOA effect was also significant, with faster RTs at SOA2 (*M* = 550 ms, *SD* = 83 ms) than at SOA1 (*M* = 571 ms, *SD* = 83 ms), *d* = 0.25, thus showing that participants responded faster at later SOAs. The significant two-way interaction between Compatibility and Clarity reflects larger compatibility effects for clear stimuli (*M* = 19 ms, *SD* = 61 ms) than for blurred stimuli (*M* = 6 ms, *SD* = 65 ms), *d* = 0.21 and BF_10_ = 0.367. Finally, the two-way interaction between Animacy and Clarity was significant, as RTs in the clear condition were similar for natural (*M* = 555 ms, *SD* = 84ms) and computer-generated stimuli (*M* = 552 ms, *SD* = 87 ms), *d* = 0.04, while RTs in the blurred condition were slower for the computer-generated stimuli (*M* = 572 ms, *SD* = 83 ms) than for the human stimuli (*M* = 563 ms, *SD* = 80ms), *d* = 0.11. Note that neither the two-way interaction between Compatibility and Animacy nor the three-way interaction between Compatibility, Animacy and Clarity were included as they did not improve model fit. See Appendix A for detailed result tables.

#### Errors

All 8,264 observations were included in the error analyses. See Appendix B (Tables B1 and B2) for the model selection procedure, the saturated model, and descriptive statistics. The final model included the main effects of Compatibility and Clarity, as well as their interaction, see Table 2. Error rates (ERs) were higher in incompatible (*M* = 8.61%, *SD* = 1.16%) than in compatible trials (*M* = 7.07%, *SD* = 1.03%), *d* = 1.40. The significant two-way interaction between Compatibility and Clarity suggest that the error compatibility effect was larger for the clear stimuli (*M* = 3.20%, *SD* = 14.07%) than for the blurred stimuli (*M* = *-*0.11%, *SD* = 12.64%), *d* = 0.25 and BF_10_ = 0.779.

**Table 2:** Final model of errors using a binomial distribution and logit link function

Fixed effect	Estimate	*SE*	t-value	p-value
**(Intercept)**	**-2.684**	**0.103**	**-26.177**	** < 2 × 10**^–16^***
**Compatibility**	**0.109**	**0.042**	**2.624**	0.009**
Clarity	-0.036	0.052	-0.690	0.490
**Compatibility x Clarity**	**-0.122**	**0.042**	**-2.927**	0.003**

*SOA* stimulus-onset asynchrony

^*^ *p* < .05, **p < .01, ***p < .001

#### Two-alternative forced choice (2AFC)

We used GLMMs and backward selection to assess the effect of Animacy, Clarity and their interaction on errors (0 vs. 1) in the 2AFC task. This task was included to ensure that differences in the SRC task were not driven by differences in the intelligibility of the effector in the distractor stimuli across conditions. See Appendix C for the model selection procedure, saturated model, and descriptive statistics (Tables C1 and C2). No main effects or interactions improved model fit, thus there was no evidence for a difference in 2AFC accuracy between Animacy and Clarity.

### Interim discussion

Experiment 1 aimed to disentangle effects of the Animacy and Clarity of the distractor stimulus in a factorial design. Results showed that Animacy did not affect compatibility effects, but results showed decreased compatibility effects for the Clarity manipulation. These results do not support predictions from the self-other overlap and motivational accounts for stimulus animacy. Experiment 2 aimed to further explore the effect of stimulus clarity on compatibility effects by establishing if systematically degrading the clarity of the human distractor stimuli affected compatibility effects parametrically. As we did not find a compatibility effect for Animacy, we did not include the computer-generated stimuli in Experiment 2.

## Experiment 2

### Methods

#### Participants

Participant recruitment was conducted on Prolific (/prolific.co) and testing through Gorilla (/gorilla.sc), using the same recruitment and inclusion criteria as in Experiment 1, with the addition that participants from Experiment 1 were excluded. Following the pre-registration (AsPredicted.org, #98,537), we followed Bayes’ stopping rule to determine sample size. We initially recruited 40 participants and calculated the BF_10_ for the interaction between Compatibility and Clarity by comparing a model with the interaction to a model without the interaction, following Jarosz and Wiley (2014). While the BF_10_ for the key interaction was between 0.2 and 3 or until BF_10_ was stable for four consecutive participants, we increased sample size by increments of two participants (one per symbol-action pairing counterbalance). We ceased testing at 70 participants, as the BF_10_ remained stable for four consecutive participants (BF_10_ = 2.718). Three participants were excluded and replaced for showing an ER of > 50% in one or more conditions of the SRC task, three were excluded and replaced for having an overall ER of > 3 SDs from the group mean, and one participant for scoring 0% on the 2AFC identification task. Participants were paid £2 for their time. The final sample consisted of 70 participants (35 female; *M*_age_ = 25.77 years, *SD*_age_ = 3.07 years, range = 20–31 years).

#### Materials

**Fig. 3:**
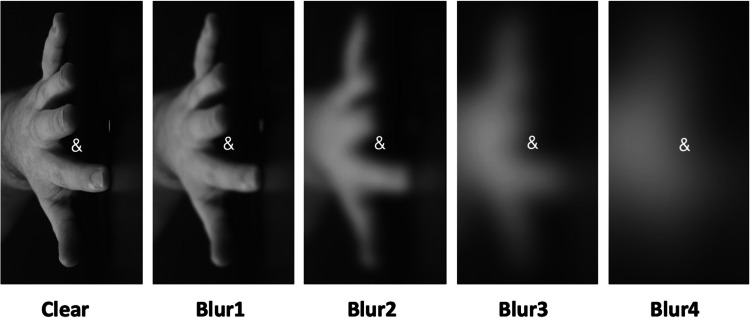
Examples of the five Clarity conditions: Clear and the four blurring levels, Blur1–4

The SRC video stimuli were identical to those in Experiment 1, except that we only used human distractors and five instead of two clarity levels. In Fig. 3, Clear refers to the condition where visual clarity was not manipulated. In Blur1, we applied Gaussian blurring at 25% in Final Cut Pro and blur boost was set to 1. For Blur2, blurring was set to 50% and boost to 2, for Blur3, blurring was set to 50% and boost to 5, for Blur4, blurring was set to 50% and blur boost to 10. The conditions Clear and Blur3 were also used in Experiment 1, as the Clear and Blurred human stimuli, respectively.

#### Procedure

The experiment procedure was identical to that of Experiment 1, except participants completed four blocks of 40 trials in the main task (2 effectors × 2 prompts × 2 SOAs × 5 Clarity conditions). The catch trials were the same as in Experiment 1, and a 2AFC identification task was also included after the SRC task, consisting of 40 trials (4 × 2 effectors × 5 Clarity levels), mixed by condition. Stimulus-symbol association was again counterbalanced across participants.

### Data processing and analysis

Responses were recorded and processed as in Experiment 1. GLMMs were again used to determine the effects of Compatibility (compatible vs. incompatible, coded as -0.5 and 0.5, respectively), SOA (SOA1 vs. SOA2, coded as -0.5 and 0.5, respectively), Clarity (-2, -1, 0 1, 2) and their interactions on raw RTs (ms) and errors (0 vs. 1). The RT models utilised the gamma distribution and identity link function, following Lo and Andrews (2015). The accuracy analyses used a binomial distribution and logit link function. The maximal converging random effect structure for the RT analyses consisted of by-participant intercepts and slopes for Clarity and SOA. For the error analyses, the maximal random effect structure consisted of by-participant intercepts and by-participant slopes for SOA. The same backward selection procedure was used as in Experiment 1.

### Results

#### RTs

The full dataset for 70 participants consisted of 11,166 observations. For the RT analyses, erroneous trials were removed (928 trials, 8.31%), including 422 wrong answers, five anticipatory responses (RT < 200 ms), and 501 late responses (RT > 1,000 ms). We further removed 738 observations with RTs outside of three MADs from each participant’s mean in each experimental condition (i.e., each combination of Compatibility, Clarity and SOA). The remaining 9,500 trials were included in the RT analyses.

**Fig. 4:**
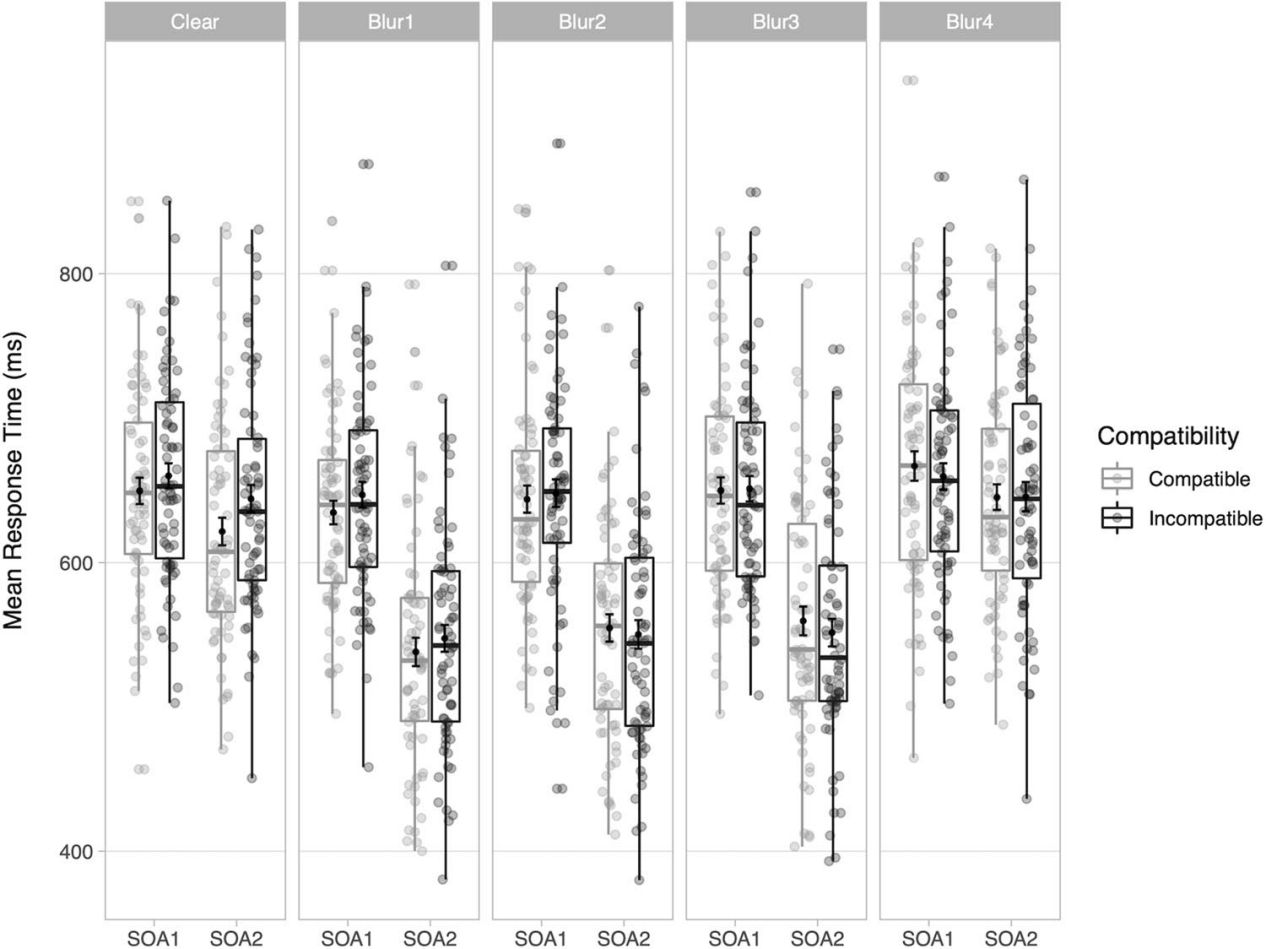
Mean response times (RTs) in milliseconds (ms) for correct trials in the stimulus–response compatibility (SRC) tasks for each experimental condition. Points in the background show the raw mean RTs for each participant (points are offset on the x-axis for clarity). The boxplots indicate the first, second (median) and third quartiles, and whiskers indicate 1.5 times the interquartile range of the distribution. Black points in the foreground show the mean and error bars indicate standard errors

The backward selection procedure for the saturated model is described in Appendix D, Table D1. The final model (Table 3) included the main effects of Compatibility, Clarity, SOA and the two-way interaction between Compatibility and Clarity. The main effect of SOA was significant, with slower RTs at SOA1 (*M* = 651 ms, *SD* = 75 ms) than at SOA2 (*M* = 585 ms, *SD* = 91 ms), *d* = 0.79. The significant main effect of Clarity revealed that RTs decreased for Blur1-3 relative to the Clear condition while RTs increased again for Blur4, but note that this result refers to the overall combined effect for Clarity, and not to the interaction between blurring and compatibility. Crucially, the two-way interaction between Compatibility and Clarity was significant with BF_10_ = 2.718, suggesting that the Compatibility effect decreased for higher levels of Gaussian blur (Blur2-4) (cf. Table 4, Fig. 4).

**Table 3:** Final model of reaction times (RTs) in milliseconds (ms) using a gamma distribution and identity link function

Fixed effect	Estimate	*SE*	t-value	p-value
**(Intercept)**	**626.724**	**3.285**	**190.772**	** < 2 × 10**^–16^***
Compatibility	2.010	1.093	1.839	0.066
**Clarity**	**3.276**	**1.166**	**2.809**	0.005**
**SOA**	**-33.170**	**1.672**	**-19.839**	** < 2 × 10**^–16^***
**Compatibility x Clarity**	**-2.659**	**0.780**	**-3.410**	** < 0.001**

*SOA* stimulus-onset asynchrony

^*^ *p* < .05, ** *p* < .01, *** *p* < .001

**Table 4:** Compatibility effects in milliseconds (ms) for each Clarity level

Clarity	*M* (ms)	*SD* (ms)
Clear	16	57
Blur1	11	58
Blur2	0	66
Blur3	-3	64
Blur4	-3	62

#### Errors

The full dataset of 11,166 observations was included in the error analyses. The backward selection procedure from the saturated model is described in Appendix E, Table E1. No main effects or interactions were found to improve model fit in Experiment 2. In other words, there was no evidence for an effect of Compatibility, Clarity, SOA or their interactions on the errors in the SRC task.

#### 2AFC

As in Experiment 1, we used GLMMs to assess the effect of Clarity on errors (0 vs. 1) in the 2AFC identification task. There was no evidence of an effect of Clarity on errors in this task (cf. Appendix F, Tables F1 and F2).

## General discussion

This study aimed to disentangle effects of two stimulus-driven manipulations, Animacy and Clarity, on automatic imitation of manual actions. We intended to evaluate whether animacy modulates automatic imitation per predictions of the self-other overlap and motivational accounts when considering the visual clarity of the distractor stimuli. We conducted two experiments in which we systematically manipulated Animacy and Clarity (Experiment 1) or parametrically modulated Clarity of the human stimuli only (Experiment 2). The results from Experiment 1 showed first that compatible trials were responded to faster (*M* = 554 ms, *SD* = 83 ms) than incompatible trials (*M* = 567 ms, *SD* = 85 ms), thus showing a modest overall compatibility effect of 13 ms. Second, contrary to our predictions, Animacy did not affect compatibility effects, but reducing the clarity of the visual distractors using Gaussian blur decreased compatibility effects, with clear distractors showing an effect of 19 ms (*SD* = 61 ms) and blurred distractors showing a smaller effect of 6 ms (*SD* = 65 ms). Experiment 2 showed no overall compatibility effect, which was to be expected given the design aimed to reduce compatibility. Note that the largest compatibility effect was 16 ms for clear distractors, followed by 11 ms for Blur1, 0 ms for Blur2, and -3 ms for Blur3 and Blur4. We therefore found an overall compatibility effect for the baseline (clear) condition (albeit slightly smaller than the compatibility effect for the equivalent condition in Experiment 1 of 21 ms), but compatibility effects thus decreased with increasing blurriness levels, resulting in an overall null effect for compatibility. We also found a main effect of overall SOA (i.e., combined RTs for both compatibility conditions) in both experiments, with faster RTs for later SOAs. SOA did not interact with any of the other main effects, and decreasing RTs for later SOAs have been reported in SRC studies (Kerzel & Bekkering, 2000; Wilt et al., 2022), and are thought to be the result of the availability of additional information about the distracter stimulus as the trial unfolds. Our results thus do not conform to predictions from current theoretical accounts as animacy did not modulate automatic imitation. Instead, it appears that only clarity of the stimuli affected automatic imitation in both experiments, indicating that the blurred stimuli evoked less sensorimotor priming.

### Animacy manipulation

We found a null effect for animacy, replicating results from Oztop et al. (2005), Bird et al. (2007), Jansson et al. (2007), Liepelt et al. (2010), Kupferberg et al. (2012) and Wilt et al. (2022), but not results from Brass et al. (2001), Kilner et al. (2003) and Press et al. (2005). Our findings for animacy did not support predictions of self-other overlap and motivational theories, which both predicted enhanced automatic imitation for human stimuli. We argued that the mixed pattern in the results for past animacy studies, with some showing decreases for non-human stimuli and others showing a null effect, was due to stimulus-driven differences between the studies. Studies that used stylised non-human stimuli, or stimuli with a lower complexity, showed decreased automatic imitation for the non-human stimuli. When the stimuli for both conditions were matched, no animacy effects were found (Jansson et al., 2007). We aimed to use human and non-human stimuli that were matched in size and shape. When piloting prior to data analysis for Experiment 1, we found that the artificial hands were too difficult to distinguish from the human hands, especially in the blurred condition, when we used a matched skin-colour for both stimulus types. Therefore, to ensure that the difference between the two types of stimuli remained intact during visual degradation, we created blue computerised hands and used a post-task 2AFC task to establish whether all stimuli could be classified accurately in terms of their actions. Indeed, the 2AFC results confirmed that stimuli could be identified correctly, as performance for all stimuli was at ceiling level (> 95% correct).

However, our stimuli contained an intrinsic confound between animacy (stimulus-driven) and belief (top-down) manipulations per the status of the stimuli: our stimulus-driven animacy status manipulation may also have invoked a belief manipulation. Several studies aimed to disentangle belief manipulations and stimulus-driven manipulations (Gowen et al., 2016; Liepelt & Brass, 2010; Press et al., 2006; Stanley et al., 2007). However, while some studies (Gowen & Poliakoff, 2012; Longo et al., 2008) report effects of belief manipulations, others report null effects (Liepelt & Brass, 2010; Press et al., 2006). Cracco et al.’s (2018) meta-analysis also found no effect of belief manipulations on automatic imitation. At this stage, the number of studies investigating belief manipulation is overall too low to reach definite conclusions regarding their effect on automatic imitation. Cracco et al. also suggested that compatibility effects do not differ when the non-human stimuli are too human-like, as was likely confirmed by our results.

In our study design we aimed to minimise this intrinsic confound between animacy and the associated belief manipulation in two ways. First, we ensured that the non-human stimuli were distinguishable from the human stimuli, as they were coloured blue and did not have fingernails or skin folds or texture. Second, we informed participants that the non-human distractors were computer-generated in the description of the experiment on prolific, and in the task instructions. However, our Experiment 1 did not include a rating procedure in which participants judged the stimuli in terms of their animacy. We conducted a separate animacy rating task online with 20 participants not included in either experiment, in which we asked them to rate the four stimuli from Experiment 1 as human or non-human on a scale between 0 and 100 (0 = human, 100 = artificial). The results showed that participants gave the clear human stimuli a rating of 6.96 (SD = 14.1), the blurry human stimuli 9.82 (SD = 14.3), the clear computer-generated stimuli 92.5 (SD = 14.5), and the blurry computer-generated stimuli 90.3 (SD = 15.0). A two-way ANOVA on these ratings showed that the human and computer-generated ratings differed significantly (*F*(3,1316) = 3,635, *p* < 0.001), with no effect of Clarity and no interactions. Planned t-tests showed no difference between the clear and blurry human stimuli and between the clear and blurry computer-animated stimuli and a difference in mean rating between both human types of stimuli and the computer-generated stimuli. Interestingly, these results indicate that clarity reduction through blurring of the stimuli did not affect perceived stimulus animacy. Moreover, it appears that the confound between belief and stimulus-driven manipulation did not affect our results, as we report a null effect for animacy in Experiment 1. Thus, our study provides further evidence that human actions do not hold a privileged status compared to non-human actions due to the observer’s greater familiarity or affiliation with human-generated actions.

### Clarity manipulation

Results from Experiment 1 for the Clarity manipulation showed a significant effect, with smaller compatibility effects for blurred (6 ms) than for clear (19 ms) stimuli. The results from Experiment 2 strengthened the evidence for the trend found for clarity in Experiment 1. The clear and Gaussian blur conditions used in Experiment 1 were identical to the Clear and Blur3 conditions in Experiment 2, respectively. The compatibility effect for the Clear condition in Experiment 1 for the human condition was 19 ms, while the effect for the Clear condition in Experiment 2 was 16 ms. The compatibility effect for the Blurred condition in Experiment 1 for the human condition was 6 ms, while the effect for the Blur3 condition in Experiment 2 was -3 ms. Therefore, Experiment 2 replicated the pattern in the results for these two identical stimulus conditions but the difference between the two conditions was larger for Experiment 2 (19 ms for Experiment 1 and 13 ms for Experiment 2). Also, the compatibility effect disappeared in Experiment 2 for the two conditions with the highest level of Gaussian blur (Blur3-4), with slightly faster RTs for the compatible than incompatible conditions. Experiment 2 thus showed that parametric degradation of the visual clarity results in gradual smaller compatibility effects. At a relatively mild level of blurring (Blur1), a compatibility effect was still evoked (11 ms), but this effect disappeared once more Gaussian blur was added.

Results for both experiments demonstrated that clarity effects on automatic imitation were elicited by a reduction of stimulus clarity and associated sensorimotor priming, occurring in the absence of reduced identification of the blurred stimuli in the 2AFC tasks. Both 2AFC tasks reported a ceiling effect, with no effects of either Animacy (Experiment 1) or Clarity (Experiments 1 and 2) on accuracy. Human processing of (the mental imagery of) biological movement is incredibly robust (Casile & Giese, 2005) even with impoverished information (e.g., point light displays; Johansson, 1973, 1976). Moreover, it has been proposed that perceptual processes, such as identification of visual stimuli in an 2AFC task, relies on different cognitive and neural systems from visuomotor processing (Goodale & Milner, 1992). Therefore, general motion detection ability paired with participants’ prior experience with the stimuli during the SRC task may explain participants’ high level of accuracy during the classification task. The number of errors was at 2.55% (*SD* = 6.78%) for the clear stimuli, 2.86% (*SD* = 6.08%) for Blur1, 1.99% (*SD* = 4.65%) for Blur2, 1.96% (*SD* = 5.05%) for Blur3, and 4.26% (*SD* = 10.73%) for Blur4. These results imply that automatic imitation does not depend on the extent to which compatible and incompatible distractors can be distinguished in a direct comparison. It instead appears that the features that upon observation engage associated action motor patterns need to be clearly observable to evoke compatibility effects.

### Limitations and future directions

Our study’s main limitation was the initial low sample size target for Experiment 1. We set this target based on our previous study using a comparable design with speech stimuli (Wilt et al., 2022). However, the sample size of 32 participants was insufficient to run the intended analyses. We deviated from our preregistration and doubled the sample size. We estimate that the possible low power for Experiment 1 could be due to two factors. First, there were significant differences in the participant recruitment procedure for Wilt et al. (2022) and the current study. Because Wilt et al. used a speech SRC paradigm, each participant completed an eligibility test to ensure they had a microphone and headphones of sufficient quality for completing the study. As our study did not require the recording of speech, nor did it require participants to have a headphone setup, we decided to not pre-screen our participants with a similar eligibility test. Nevertheless, by increasing the number of participants we were able to run all our planned analyses satisfactorily.

A similar issue was likely the case for Experiment 2. Here, we based our minimum sample size on a previous experiment (i.e., Experiment 1) and set it to 64 participants using a similar experimental design and setup. However, we needed to test additional participants beyond the minimum number before the BF_10_ became stable, per our preregistration. We found a compatibility effect (16 ms and -3 ms for conditions Clear and Blur3) that largely replicated the effect we report for the same conditions in Experiment 1. However, the BF_10_ was not conclusive at 2.718 (our threshold was set to BF_10_ > 3) for the relevant interaction between Compatibility and Clarity, even though inclusion of the interaction significantly benefitted model fit.

Moreover, compatibility effects for both studies were on the low side (13 ms for Experiment 1, and no effect for Experiment 2) compared to previously published studies. These compatibility effects might have been due to our decision to run both studies via an online platform, although others find larger compatibility effects in online settings (Genschow et al., 2022). The variability in our experiment was overall high, which might have reduced the overall effect.

We mentioned above that the compatibility effect decreased in both experiments for Gaussian blur stimulus conditions in the absence of a decrease of discriminability between the two actions (index and middle finger movement) as measured using an 2AFC task. Future research could explore this relationship further, for example, by using actions in which the depicted action is visually or aurally ambiguous. This could be done, for instance, by using manual actions that are perceived as intermediate between the two actions. A design could be used where each participant first completes a pre-test to establish which stimuli are least and most ambiguous. These stimuli could then be deployed in a factorial design that aims to disentangle action discriminability and the extent to which a depicted action can evoke compatibility effects. This manipulation could be combined with stimuli that vary in how human-like in origin they appear (e.g., by presenting images morphed between a human hand and a silhouette or stylised hand).

The results from both experiments showed that severely degrading the stimulus materials did not significantly affect participants’ ability to classify the type of movement, but that degraded stimuli still evoked less sensorimotor priming than the clear stimuli. This dissociation between the results of the two tasks suggests that stimulus classification and sensorimotor priming engage different cognitive processes, with stimulus classification potentially relying more on processes associated with visual perception of stimulus characteristics and visuomotor priming engaging motor patterns. Further studies could build on this finding by further elucidating the spatial and temporal neural signature of the combination of both processes, for example, using electroencephalography (EEG), or functional magnetic resonance imaging (fMRI), as these methods could be used to outline the neural bases of either process.

Our study aimed foremost to disentangle effects of stimulus animacy and different levels of stimulus clarity, but our results also shed light on potential mechanisms that are involved in animacy and clarity processing. For instance, our results demonstrated that systematically evaluating factors that decrease sensorimotor priming can enhance our understanding of the mechanisms supporting automatic imitation of observed actions. Our results show that human actions and matched non-human actions as used in our Experiment 1 may serve as a baseline for sensorimotor priming. Any stimulus that is degraded sufficiently evoked less priming and thus less automatic imitation. Future studies could focus on factors that reduce sensorimotor priming and include top-down as well as stimulus-driven factors. For instance, Genschow et al. (2015) conducted a test of the self-overlap account by manipulating participants’ focus on similarities or differences between theirs and the agent’s hand, while keeping the stimulus material constant, thus implementing a top-down or belief manipulation. They found that focusing on similarities, as compared with differences, increased automatic imitation, and that focusing on differences decreased automatic imitation. They therefore demonstrated that attentional focus manipulations can affect automatic imitation. Moreover, they showed that, instead of similarity enhancing automatic imitation, as predicted by the self-other overlap and motivational accounts, dissimilarity decreased automatic imitation. Our results fit with those of Genschow et al., except we employed stimulus-driven manipulations. We expect that systematic evaluation of top-down and stimulus factors modulating automatic imitation will further contribute to theory development.

## Conclusions

In conclusion, automatic imitation was not modulated by animacy, but reducing how much a distractor evokes sensorimotor priming, as implemented by visual degradation in our study, reduced automatic imitation. Our results do not support the self-other and motivational accounts of automatic imitation. Future work should focus on further identifying how stimulus-driven (complexity, clarity) and top-down (e.g., focused attention or belief manipulations) factors, or a combination of both factors, evoke sensorimotor priming.

## Supplementary information

Below is the link to the electronic supplementary material.

Supplementary file1 (DOCX 37 KB)

## Data Availability

The aims, predictions, design, and proposed analysis of the experiment were preregistered on https://www.AsPredicted.org as 98,537 ‘The effect of stimulus clarity on automatic imitation of manual actions’ (https://aspredicted.org/d3kf9.pdf) and 92,608 ‘Automatic imitation of synthetic and unclear hand movements’ (https://aspredicted.org/bk642.pdf). All stimulus materials and raw (text) data can be found on the Open Science Framework at: https://osf.io/45hje/

## Code availability

All R scripts can be found on the Open Science Framework at: https://osf.io/45hje/
