# Supplementary material for: Automatic imitation is modulated by stimulus clarity but not by animacy
Source: Atten Percept Psychophys. 2024 Jul 31;86(6):2078–92. doi: 10.3758/s13414-024-02935-1 (PMC11411005; doi:10.3758/s13414-024-02935-1)
Supplement: Supplementary file 1 — Supplementary file1 (DOCX 37 KB) [file 13414_2024_2935_MOESM1_ESM.docx]

SUPPLEMENTARY MATERIALS

**Appendix A**. Saturated model and backward selection process for the RT analyses in Experiment 1.

The maximal random effect structure to converge and pass singularity checks included by-participant random intercept and no slopes. Starting with the saturated model (Table A1), we performed backward selection to determine the best fitting model. The four-way interaction between Compatibility, Animacy, Clarity, and SOA did not benefit model fit and was removed from the model (χ^2^(1)=2.077, *p*=0.150, BF_10_=0.050). None of the three-way interactions benefitted model fit and were removed in the following order: Compatibility x Animacy x Clarity (χ^2^(1)=0.850, *p*=0.357, BF_10_=0.018); Compatibility x Animacy x SOA (χ^2^(1)=1.354, *p*=0.245, BF_10_=0.018); Animacy x Clarity x SOA (χ^2^(1)=2.135, *p*=0.144, BF_10_=0.030); Compatibility x Clarity x SOA (χ^2^(1)=2.815, *p*=0.093, BF_10_=0.050). Next, we removed the following two-way interactions in order: Clarity x SOA (χ^2^(1)=0.069, *p*=0.794, BF_10_=0.018); Compatibility x Animacy (χ^2^(1)=0.115, *p*=0.734, BF_10_=0.011); Animacy x SOA (χ^2^(1)=2.111, *p*=0.146, BF_10_=0.030); Compatibility x SOA (χ^2^(1)=2.86, *p*=0.091, BF_10_=0.050). The two-way interaction between Compatibility and Clarity benefited model fit (χ^2^(1)=6.196, *p*=0.013, BF_10_=0.368), as did the two-way interaction between Animacy and Clarity (χ^2^(1)=7.556, *p*=0.006, BF_10_=0.607). Note that the low BF_10_ values for these interactions suggest that evidence for either interaction is noisy. The main effect of SOA improved model fit and was kept in the model (χ^2^(1)=82.775, *p*=2.2x10^-6^, BF_10_=1.172x10^16^). The final model included the main effects of Compatibility, Clarity, Animacy, SOA as well as the two-way interaction between Compatibility and Clarity, and the two-way interaction between Clarity and Animacy.

**Table A1.** Saturated model for the reaction time (RT) analyses.

| Fixed Effect | Estimate | *SE* | t-value | p-value |
| --- | --- | --- | --- | --- |
| **(Intercept)** | **572.589** | **3.803** | **150.552** | **<2 x 10^-16^***** |
| **Compatibility** | **5.633** | **1.049** | **5.368** | **7.94 x 10^-8^***** |
| **Animacy** | **1.341** | **1.072** | **1.251** | **0.211** |
| **Clarity** | **7.273** | **1.060** | **6.864** | **6.70 x 10^-10^***** |
| **SOA** | **-10.159** | **1.064** | **-9.549** | **<2 x 10^-16^***** |
| Compatibility x Animacy | 0.441 | 1.055 | 0.418 | 0.676 |
| **Compatibility x Clarity** | **-2.769** | **1.056** | **-2.624** | **0.009**** |
| **Animacy x Clarity** | **3.089** | **1.047** | **2.950** | **0.003**** |
| Compatibility x SOA | -1.860 | 1.062 | -1.751 | 0.080 |
| Animacy x SOA | 1.554 | 1.066 | 1.458 | 0.145 |
| Clarity x SOA | 0.327 | 1.059 | 0.309 | 0.758 |
| Compatibility x Animacy x Clarity | 1.075 | 1.060 | 1.015 | 0.310 |
| Compatibility x Animacy x SOA | -1.318 | 1.061 | -1.241 | 0.215 |
| Compatibility x Clarity x SOA | 1.821 | 1.057 | 1.723 | 0.085 |
| Animacy x Clarity x SOA | -1.635 | 1.056 | -1.548 | 0.122 |
| Compatibility x Animacy x Clarity x SOA | -1.589 | 1.069 | -1.487 | 0.137 |
| *Note:* SOA= Stimulus-Onset Asynchrony. * *p*<.05, ***p*<.01, ***p<.001. | | | | |

**Table A2.** Mean reaction times (RTs) in milliseconds (ms) and standard deviations (*SD*s) per condition.

| Animacy | Clarity | SOA | Compatibility | *M* (ms) | *SD* (ms) |
| --- | --- | --- | --- | --- | --- |
| Human | Clear | SOA1 | Compatible | 552 | 76 |
|  |  |  | Incompatible | 585 | 89 |
|  |  | SOA2 | Compatible | 537 | 84 |
|  |  |  | Incompatible | 546 | 80 |
|  | Blurred | SOA1 | Compatible | 574 | 74 |
|  |  |  | Incompatible | 571 | 80 |
|  |  | SOA2 | Compatible | 550 | 85 |
|  |  |  | Incompatible | 557 | 81 |
| Computer-Generated | Clear | SOA1 | Compatible | 548 | 79 |
|  |  |  | Incompatible | 573 | 92 |
|  |  | SOA2 | Compatible | 540 | 90 |
|  |  |  | Incompatible | 550 | 85 |
|  | Blurred | SOA1 | Compatible | 574 | 85 |
|  |  |  | Incompatible | 591 | 86 |
|  |  | SOA2 | Compatible | 560 | 82 |
|  |  |  | Incompatible | 564 | 76 |

*Note:* SOA = Stimulus-Onset Asynchrony.

**Appendix B**. Saturated model and backward selection process for the error analyses

The maximal random effect structure to converge and pass singularity checks included by-participant random slopes for Clarity. We performed backward selection to determine the best fitting model, starting with the saturated model (Table B1). The four-way interaction between Compatibility, Animacy, Clarity, and SOA did not benefit model fit and was excluded from the model (χ^2^(1)=0.099, *p*=0.753, BF_10_=0.012). None of the three-way interactions benefitted model fit and were removed in the following order: Compatibility x Animacy x SOA (χ^2^(1)=0.017, *p*=0.894, BF_10_=0.011); Compatibility x Animacy x Clarity (χ^2^(1)=0.082, *p*=0.775, BF_10_=1.37 x 10^-5^); Compatibility x Clarity x SOA (χ^2^(1)=0.125, *p*=0.724, BF_10_=0.012); Animacy x Clarity x SOA (χ^2^(1)=0.131, *p*=0.717, BF_10_=0.012). Next, we removed the following two-way interactions in order: Compatibility x Animacy (χ^2^(1)= 0.258, *p*=0.612, BF_10_=0.013); Animacy x SOA (χ^2^(1)=0.318, *p*=0.573, BF_10_=0.013); Clarity x SOA (χ^2^(1)=0.484, *p*=0.487, BF_10_=0.014); Compatibility x SOA (χ^2^(1)=0.973, *p*=0.324, BF_10_=0.018), Animacy x Clarity (χ^2^(1)=1.271, *p*=0.260, BF_10_=0.020). The two-way interaction between Compatibility and Clarity was found to improved model fit and was kept in the model (χ^2^(1)=8.396, *p*=0.004, BF_10_=0.779). Note that the low BF_10_ suggests that evidence for this interaction is noisy. The main effect of Animacy did not benefit model fit and was removed from the model (χ^2^(1)=0.047, *p*=0.828, BF_10_=0.012), as was the main effect of SOA (χ^2^(1)=0.630, *p*=0.013, BF_10_=0.012).

**Table B1.** Saturated model for the error analyses in Experiment 1.

| Fixed Effect | Estimate | *SE* | t-value | p-value |
| --- | --- | --- | --- | --- |
| **(Intercept)** | **-2.687** | **0.103** | **-26.180** | **< 2 x 10-^16^***** |
| **Compatibility** | **0.109** | **0.042** | **2.619** | **0.009**** |
| Animacy | 0.006 | 0.042 | 0.145 | 0.885 |
| Clarity | -0.036 | 0.052 | -0.689 | 0.491 |
| SOA | -0.025 | 0.042 | -0.594 | 0.522 |
| Compatibility x Animacy | 0.022 | 0.042 | 0.518 | 0.604 |
| **Compatibility x Clarity** | **-0.120** | **0.042** | **-2.889** | **0.004**** |
| Animacy x Clarity | -0.043 | 0.042 | -1.028 | 0.304 |
| Compatibility x SOA | 0.038 | 0.042 | 0.914 | 0.361 |
| Animacy x SOA | 0.021 | 0.042 | 0.509 | 0.611 |
| Clarity x SOA | -0.027 | 0.042 | -0.649 | 0.516 |
| Compatibility x Animacy x Clarity | -0.012 | 0.042 | -0.294 | 0.769 |
| Compatibility x Animacy x SOA | 0.006 | 0.042 | 0.133 | 0.894 |
| Compatibility x Clarity x SOA | -0.014 | 0.042 | -0.347 | 0.723 |
| Animacy x Clarity x SOA | 0.017 | 0.042 | 0.417 | 0.677 |
| Compatibility x Animacy x Clarity x SOA | -0.013 | 0.042 | -0.319 | 0.750 |
| *Note:* SOA= Stimulus-Onset Asynchrony. * *p*<.05, ***p*<.01, ***p<.001 | | | | |

**Table B2.** Mean error rates in percentages and standard deviations (*SD*s) per condition.

| Animacy | Clarity | SOA | Compatibility | *M* (%) | *SD* (%) |
| --- | --- | --- | --- | --- | --- |
| Human | Clear | SOA1 | Compatible | 6.37 | 9.42 |
|  |  |  | Incompatible | 8.54 | 12.54 |
|  |  | SOA2 | Compatible | 5.96 | 7.69 |
|  |  |  | Incompatible | 9.04 | 11.38 |
|  | Blurred | SOA1 | Compatible | 9.04 | 10.38 |
|  |  |  | Incompatible | 8.35 | 12.84 |
|  |  | SOA2 | Compatible | 7.40 | 9.15 |
|  |  |  | Incompatible | 7.50 | 11.00 |
| Computer-Generated | Clear | SOA1 | Compatible | 6.73 | 9.90 |
|  |  |  | Incompatible | 9.45 | 13.27 |
|  |  | SOA2 | Compatible | 6.02 | 9.78 |
|  |  |  | Incompatible | 10.85 | 11.33 |
|  | Blurred | SOA1 | Compatible | 7.69 | 10.53 |
|  |  |  | Incompatible | 7.55 | 9.15 |
|  |  | SOA2 | Compatible | 7.31 | 10.34 |
|  |  |  | Incompatible | 7.58 | 11.12 |

*Note:* SOA = Stimulus-Onset Asynchrony.

**Appendix C**. Saturated model, backward selection and descriptive statistics process for the two-alternative forced choice (2AFC) error analyses in Experiment 1.

The maximal random effect structure to converge and pass singularity checks included only by-participant intercepts and slopes for Animacy. The saturated model is displayed in Table C1). The two-way interaction between Animacy and Clarity failed to improve model fit and was removed from the model (χ^2^(1)=0.519, *p*=0.471, BF_10_=0.029), followed by the main effects of Clarity (χ^2^(1)=0.762, *p*=0.382, BF_10_=0.032) and Animacy (χ^2^(1)=2.005, *p*=0.157, BF_10_=0.060). Thus, there was no evidence for an effect of Animacy, Clarity or of their interaction on errors in the 2AFC identification task (cf. Table C2).

**Table C1.** Saturated model for the error analyses for the 2AFC task in Experiment 1.

| Fixed Effect | Estimate | | *SE* | | t-value | p-value |
| --- | --- | --- | --- | --- | --- | --- |
| **(Intercept)** | | **-4.456** | | **0.345** | **-12.865** | **< 2 x 10-^16^***** |
| Clarity | | 0.213 | | 0.292 | 0.731 | 0.465 |
| Animacy | | -0.799 | | 0.631 | -1.267 | 0.205 |
| Clarity x Animacy | | -0.420 | | 0.583 | -0.720 | 0.471 |
| * *p*<.05, ***p*<.01, ***p<.001 | | | | | | |

**Table C2**. Mean percentage error rates and standard deviation for each condition in the two-alternative forced-choice (2AFC) identification task.

| Animacy | Clarity | *M* (%) | *SD* (%) |
| --- | --- | --- | --- |
| Human | Clear | 5.50% | 2.50% |
|  | Blurred | 9.84% | 3.68% |
| Computer-Generated | Clear | 5.66% | 2.12% |
|  | Blurred | 5.66% | 2.12% |

**Appendix D**. Saturated model and backward selection process for the RT analyses in Experiment 2.

The maximal random effect structure to converge and pass singularity checks included by-participant random intercepts and no random slopes. Starting with the saturated model (Table E1), we performed backward selection to determine the best fitting model. The three-way interaction between Compatibility, Clarity, and SOA failed to benefit model fit and was removed from the model (χ^2^(1)= 0.634, *p*=0.426, BF_10_=0.018). The two-way interaction between Compatibility and SOA failed to improve model fit (χ^2^(1)=0.067, *p*=.795, BF_10_=0.011), as did the two-way interaction between Clarity and SOA (χ^2^(1)=0.652, *p*=0.419, BF_10_=0.011). The two-way interaction between Compatibility and Clarity improved model fit and was kept in the model (χ^2^(1)=11.09, *p*=0.0009, BF_10_=2.718), as was the main effect of SOA (χ^2^(1)=80.569, *p* < 2 x 10^-16^, BF_10_=4.33 x 10^15^). The final model included the main effects of Compatibility, Clarity, SOA as well as the two-way interaction between Compatibility and Clarity.

**Table D1.** Saturated model for the reaction time (RT) analyses in Experiment 2.

| Fixed Effect | Estimate | *SE* | t-value | p-value |
| --- | --- | --- | --- | --- |
| **(Intercept)** | **626.715** | **3.631** | **172.594** | **< 2 x 10^-16^***** |
| Compatibility | 4.105 | 2.174 | 1.888 | 0.059 |
| **Clarity** | **3.175** | **1.160** | **2.737** | **0.006**** |
| **SOA** | **-66.284** | **2.861** | **-23.167** | **< 2 x 10^-16^***** |
| **Compatibility x Clarity** | **-5.110** | **1.499** | **-3.410** | **0.0007***** |
| Compatibility x SOA | -1.304 | 4.116 | -0.317 | 0.751 |
| Clarity x SOA | 1.302 | 1.534 | 0.848 | 0.396 |
| Compatibility x Clarity x SOA | -2.575 | 2.680 | -0.961 | 0.337 |
| *Note:* SOA= Stimulus-Onset Asynchrony. * *p*<.05, ***p*<.01, ***p<.001 | | | | |
|  | | | | |

**Appendix E**. Saturated model and backward selection process for the error analyses in Experiment 2.

The maximal random effect structure to converge and pass singularity checks included by-participant random slopes for SOA. Starting with the saturated model (Table F1), we performed backward selection to determine the best fitting model. The three-way interaction between Compatibility, Clarity, and SOA failed to benefit model fit and was removed from the model (χ^2^(1)=1.108, *p*=0.293, BF_10_= 0.017). None of the two-way interactions improved model fit, and they were removed in the following order: Clarity x SOA (χ^2^(1)=0.111, *p*=0.739, BF_10_=0.010), Compatibility x SOA (χ^2^(1)=2.152, *p*=0.142, BF_10_=0.027), Compatibility x Clarity (χ^2^(1)=2.889, *p*=0.089, BF_10_=0.041). None of the main effects improved model fit: SOA (χ^2^(1)=0.617, *p*=0.432, BF_10_=0.013), Clarity (χ^2^(1)=0.733, *p*=0.392, BF_10_=0.014), Compatibility (χ^2^(1)=2.712, *p*=0.010, BF_10_=0.014). Hence there was no evidence for an effect of Compatibility, Clarity, SOA, or their interactions on errors in the SRC task.

**Table E1.** Saturated model for the error analyses in Experiment 2.

| Fixed Effect | Estimate | *SE* | t-value | p-value |
| --- | --- | --- | --- | --- |
| **(Intercept)** | **-2.645** | **0.102** | **-26.034** | **< 2 x 10-^16^***** |
| Compatibility | 0.122 | 0.070 | 1.754 | 0.079 |
| Clarity | 0.022 | 0.025 | 0.889 | 0.374 |
| SOA | -0.071 | 0.088 | -0.807 | 0.420 |
| Compatibility x Clarity | -0.082 | 0.049 | -1.670 | 0.095 |
| Compatibility x SOA | 0.200 | 0.139 | 1.437 | 0.151 |
| Clarity x SOA | -0.012 | 0.049 | 0.390 | 0.696 |
| Compatibility x Clarity x SOA | 0.105 | 0.099 | 1.065 | 0.287 |
| *Note:* SOA= Stimulus-Onset Asynchrony. * *p*<.05, ***p*<.01, ***p<.001 | | | | |

**Appendix F**. Generalised linear mixed model (GLMM) table and descriptive statistics for errors in the two-alternative forced choice (2AFC) in Experiment 2.

The maximal random effect structure to converge and pass singularity checks included by-participant random intercepts and slopes for Clarity. The saturated model is displayed in Table F1. The main effect of Clarity failed to benefit model fit (χ^2^(1)=0.218, *p*=0.641, BF_10_=0.021). Descriptive statistics are provided in Table F2.

**Table F1.** Full model for the error analyses for the 2AFC task in Experiment 2.

| Fixed Effect | Estimate | | *SE* | | t-value | p-value |
| --- | --- | --- | --- | --- | --- | --- |
| **(Intercept)** | | **-3.955** | | **0.345** | **-11.476** | **< 2 x 10-^16^***** |
| Clarity | | -0.068 | | 0.146 | -0.465 | 0.642 |
| *Note:* SOA= Stimulus-Onset Asynchrony. Baseline condition: clear. * *p*<.05, ***p*<.01, ***p<.001 | | | | | | |

**Table F2**. Mean percentage error rates and standard deviation for each Clarity level in the two-alternative forced-choice (2AFC) identification task in Experiment 2.

| Clarity | *M* (%) | *SD* (%) |
| --- | --- | --- |
| Clear | 2.55 | 6.78 |
| Blurred Level 1 | 2.86 | 6.08 |
| Blurred Level 2 | 1.99 | 4.66 |
| Blurred Level 3 | 1.96 | 5.05 |
| Blurred Level 4 | 4.26 | 10.73 |
